# Sudanese refugees in Chad: addressing overwhelming mental health needs through sustainable partnerships

**DOI:** 10.1192/bjo.2025.10970

**Published:** 2026-02-04

**Authors:** Peter Ventevogel, Eric-Didier K. N’Dri, Ernest A. Djogo

**Affiliations:** Public Health Unit, Sustainable Responses Service, United Nations High Commissioner for Refugees, Geneva, Switzerland; Emergency, Preparedness and Response Department, World Health Organization, N’djamena, Chad; Field Office Farchana, United Nations High Commissioner for Refugees, Farchana, Chad

**Keywords:** Humanitarian emergencies, refugees, sustainable responses, Chad, Sudan

## Abstract

Since 2023, the armed conflict in Sudan has displaced nearly 900 000 people into eastern Chad, adding to pre-existing refugee populations and placing immense strain on already fragile health and social systems. Sudanese refugees experience high levels of psychological distress, yet Chad’s mental health services remain rudimentary, characterised by severe shortages of trained professionals and fragmented service provision. Despite underfunding, humanitarian agencies have explicitly prioritised mental health within their response framework, integrating mental health support into primary care and community-led initiatives. Cultural idioms of distress, stigma and language barriers continue to complicate care delivery, while simultaneously underscoring the importance of locally grounded approaches. Sustainable progress will require closer integration between humanitarian and development efforts, the strengthening of national systems and the expansion of community capacity. Innovative partnerships such as the Greentree Acceleration Plan offer pathways for scalable, culturally relevant interventions that may ultimately strengthen mental health systems for both refugees and host populations in Chad.

**Figure:**
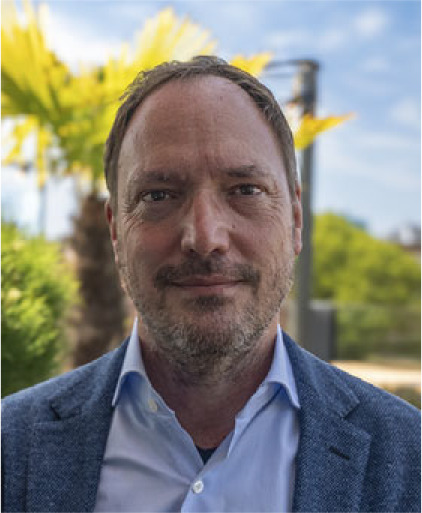

Since April 2023, violent conflict in Sudan has forced nearly 900 000 people to flee from Darfur to neighbouring Chad, where approximately 400 000 Sudanese refugees from earlier waves of displacement had already been living for several years, largely dependent on humanitarian assistance.^1^ This influx has placed immense pressure on host communities and health and social services in an already severely underserved part of the country. Sudanese refugees in Chad experience substantial levels of psychological distress, with symptoms of depression, anxiety and post-traumatic stress being widespread. This is related to high levels of exposure to violence, including sexual violence, and pervasive losses. It is further exacerbated by the ongoing hardships of daily life in refugee settlements characterised by insufficient food, inadequate shelter, limited access to basic services and uncertainty about the future.^2^

## Lack of mental health services

Despite government-led initiatives and the establishment of a National Mental Health Programme in 1998 to provide mental health services to the population and refugees, Chad’s national mental health system remains rudimentary, with a critical shortage of mental health specialists.^3^ In fact, since 2019, Chad has had no national psychiatrist in the country, and medical and nursing training contains minimal mental health content.^4^ Over two decades, since the first wave of Sudanese refugees arrived in eastern Chad, mental healthcare in refugee camps has focused primarily on integrating mental health into primary care and developing community-based psychosocial support.^5^ In 2015, the United Nations High Commissioner for Refugees (UNHCR) and partners trained hundreds of medical and other staff in the identification and management of priority conditions introduced with the World Health Organization Mental Health Gap Action Programme (mhGAP) Humanitarian Intervention Guide for the clinical management of mental, neurological and substance use conditions in humanitarian emergencies.^6^ Psychosocial services were also established by various non-governmental organisations (NGOs), often within the context of community services, child protection and programmes addressing gender-based violence.^7^ Despite two decades of efforts to integrate mental health and psychosocial support (MHPSS) into refugee services in eastern Chad, the scale of need now far outstrips available resources. Services remain fragmented and under-resourced, with unreliable supply of essential psychotropic drugs and inefficient referral systems. Consequently, the burden of care falls on non-specialist staff and refugee volunteers, often without adequate supervision or support.^8^

## Cultural explanations

Effective responses must be relevant to the people they are meant to serve, particularly when cultural idioms of distress are not readily understood by health workers. Sudanese refugees frequently use expressions such as *hozun* (deep sadness), *wajara galip* (pain in the heart) or *majnun* (madness) to articulate suffering,^9^ which can lead to misunderstandings when seeking help.^10^ Cultural explanations of mental illness are often linked to spiritual or social transgressions, shaping attitudes towards help-seeking and, at times, resulting in harmful practices, such as chaining people with psychosis or applying ritual burns to the skin to expel evil spirits.^8^ Language barriers further complicate the provision of appropriate care. Refugees from Darfur speak Sudanese Arabic, which differs markedly from Chadian Arabic. Communities on both sides of the Chad–Darfur border speak similar languages such as Masalit and Zaghawa, which are rarely spoken by humanitarian service staff, who are predominantly French-speaking.^11^

## Building the response

Despite the overwhelming need, and the fact that only 30% of the required humanitarian funding for the Sudanese refugee situation in Chad was secured in 2024, important initiatives have been launched to support the mental health and psychosocial well-being of refugees and host communities. UNHCR, the World Health Organization (WHO) and various NGOs have invested in the development of multi-layered mental health support for Sudanese refugees.^12,13^ Since 2024, WHO has reinforced its support to the National Mental Health Programme through targeted technical coaching aimed at strengthening governmental leadership in coordination, monitoring, supervision and advocacy for resource mobilisation. According to the Ajala coordination platform for humanitarian assistance, 24 organisations are currently involved in providing mental health and psychosocial support in eastern Chad (https://new.ajala.app/3w, accessed 21 October 2025).

Some of the collective initiatives to support mental health will now be described.

### Promoting intersectoral collaboration

In 2021, the Ministry of Public Health set up a Technical Working Group on MHPSS, bringing together government departments, WHO, other United Nations agencies and NGOs. At the same time, MHPSS Technical Working Groups were formed in the eastern provinces. To foster the integration of mental health and psychosocial care within various humanitarian response sectors, several 2-day workshops on the Minimum Service Package for MHPSS were organised.^14^ As a result, projects in protection, education and livelihoods began to incorporate psychosocial care.^15^ For example, UNHCR and the Red Cross of Chad collaborated in the project *Amana Wa Salama* (Peace and Security) to provide legal and practical support to refugees who had experienced torture, sexual and gender-based violence, and other human rights abuses – within both Sudan and in Chad. The project deployed psychologists to provide direct support to survivors and trained staff in responding to people in distress. Another initiative is the inclusion of social and emotional learning in refugee education, funded through the global fund Education Cannot Wait (https://www.educationcannotwait.org/). Teachers in the refugee settings are trained to adapt their teaching methods to be more responsive to the needs of refugee children who may have experienced multiple displacements, traumatic events and losses.

### Integration of mental health into primary care

The International Rescue Committee, with support from the WHO and UNHCR, has trained and supervised general practitioners, nurses and midwives to provide basic psychiatric care to refugees and host communities.^2,16^ During the first 9 months of 2025, 34 639 consultations for mental, neurological and substance use conditions were provided in primary health facilities at the refugee sites (personal communication, Dr Harouna Iname, Senior Public Health officer, UNHCR N’djamena, 6 November 2025). This exceeds the total of 29 550 consultations recorded for the calendar year 2024.^13^ Among these consultations, almost half (48%) were for epilepsy, 29% for psychotic disorders, 15% for emotional disorders (including depression, anxiety and post-traumatic stress disorder), 5% for intellectual disability and 3% for substance use disorders (data from Refugee Health Information System – https://his.unhcr.org – accessed 9 December 2025).

### Supporting community responses

UNHCR supports a network of refugee-led organisations, some of which focus on MHPSS. For instance, in Ouaddai Province, the Sudan Volunteer Organization provided support to 31 044 Sudanese refugees (56% girls, 36% women, 9% men), focusing on those who had experienced sexual violence or were injured or had lost loved ones during the conflict.^13^ In Wadi Fira Province, Sudanese refugees received training in psychological first aid (PFA) and self-care, enabling them to train others and extend support within their communities.

### Leveraging national support

An innovative initiative by the Association of Chadian Psychologists, supported technically and financially by the WHO and other United Nations agencies, deployed 14 psychologists to Wadi Fira and Ennedi-Est Provinces in June 2025.^17^ Their activities included PFA, structured individual sessions, group discussions, support for survivors of gender-based violence and capacity-building for local social and health workers. Within the first month, 12 736 people (of whom 80% were women and girls) were reached, leading to an increase in mental health referrals to health centres. For example, at the health centre in Iridimi refugee camp, weekly consultations for mental disorders rose from 6–7 to about 40–45 per week, mainly due to increased referrals by the psychologists. Psychosocial support activities, including group sessions and community events, also helped strengthen social bonds and restore a sense of normality. Refugees learned to manage stress, express emotions and regain self-confidence.^17^

## Future plans

The examples above demonstrate that, even in resource-constrained settings, meaningful progress can be achieved. However, significant needs remain, especially in the context of further reductions in humanitarian assistance. It will be essential to provide mental health and psychosocial support in ways that are both scalable and sustainable. This will require much stronger links between humanitarian and development efforts, adopting an integrated approach to strengthen national systems of care and ensure access for refugees and host communities alike. It also means intensifying efforts to build capacity within refugee communities to deliver essential MHPSS. An encouraging development is the explicit inclusion of mental health in Connexion 2030, Chad’s National Development Plan, which calls for professional capacity-building of health workers in the treatment of people with mental health conditions.^18^ The plan also proposes actions to ensure that refugees, internally displaced persons and host communities have access to essential services for education, health, livelihoods and social protection, which represents a major step towards inclusive development.

Bold new approaches will be required, including close collaboration among government institutions, United Nations agencies, NGOs and private donors. The Greentree Acceleration Plan, launched in 2025 by the United Nations Deputy Secretary-General with support from the Wellcome Trust, represents such an approach. In Chad, this plan aims to scale up MHPSS for refugees and host communities through three main pillars: (a) strengthening the national mental health system through policy reform and workforce training; (b) scaling community-level psychological interventions such as WHO’s Problem Management Plus (PM+)^19^; and (c) enhancing psychosocial support for conflict-affected children and adolescents through structured play interventions such as Team Up.^20^

## Conclusion

Despite overwhelming needs and decades of operational evidence, MHPSS in humanitarian emergencies remain underfunded, fragmented and fragile. The situation of Sudanese refugees in Chad underscores the urgent need to prioritise MHPSS as an integral component of both humanitarian and development responses. While progress has been made through community initiatives, integration into primary care and refugee-led efforts, the scale of needs far outpaces available resources. Nonetheless, opportunities remain. By building on community resilience, integrating MHPSS within national systems and fostering innovative partnerships such as the Greentree Acceleration Plan, it is possible to deliver sustainable, culturally relevant mental healthcare.
